# Screening for cytotoxic chemical constituents from *Justicia procumbens* by HPLC–DAD–ESI–MS and NMR

**DOI:** 10.1186/s13065-018-0371-z

**Published:** 2018-01-25

**Authors:** Bo Liu, Yanfang Yang, Hongbin Liu, Zhoutao Xie, Qun Li, Meng Deng, Fangping Li, Jingling Peng, Hezhen Wu

**Affiliations:** 1 Faculty of Pharmacy, Hubei University of Chinese Medicine, No.1, Huangjiahu West Road, Wuhan, 430065 China; 2 Key Laboratory of Traditional Chinese Medicine Resources and Chemistry of Hubei Province, Wuhan, 430065 People’s Republic of China; 3 Collaborative Innovation Center of Traditional Chinese Medicine of New Products for Geriatrics Hubei Province, Wuhan, 430065 China; 4 Wuhan Institute of Physics and Mathematics (WIPM) of Chinese Academy of Sciences, West No.30 Xiao Hong Shan, Wuhan, 430071 China

**Keywords:** Lignan, *Justicia procumbens*, HPLC–DAD–MS, Chemical constituents, Structural analysis, Determinate content

## Abstract

**Background:**

The Acanthaceae family is an important source of therapeutic drugs and ethno medicines. There are many famous medicinal plants from this family, such as *Andrographis paniculata*, *Baphicacanthus cusia*, and *Dicliptera chinensis*. *Justicia procumbens* (*J. procumbens*) is widely distributed in tropical and sub-tropical of the world. It has long been used in traditional Chinese medicine for cancer. The 3-(4,5-dimethylthiazol-2-yl)-2,5-diphenyltetrazolium bromide assay showed the ethyl acetate extract of *J. procumbens* had a cytotoxic activity. Therefore, qualitative and quantitative analysis of the chemical constituents in the ethyl acetate extract was important for understanding its pharmacological mechanism.

**Results:**

A high-performance liquid chromatography with diode array detection coupled to electrospray ionisation quadrupole time-of-flight tandem mass spectrometry procedure was established. Eleven dibenzylbutanes and four arylnaphthalenes were confirmed by HPLC–DAD–ESI–QTOF–MS analysis. A novel dibenzylbutane (5-methoxy-4,4′-di-*O*-methylsecolariciresinol-9′-monoacetate) and seven isomers of arylnaphthalene were isolated and characterized by NMR and QTOF–MS. Compounds 1, 2, and 13 were detected for the first time. The content of six lignans were determinated in the ethyl acetate extract.

**Conclusions:**

This study showed that the cytotoxic activity assay of *J. procumbens* could be mainly attributed to the constituents of lignans. The bioactivity of the ethyl acetate extract and determined compounds support the traditional use of this plant in cancer. These chemical constituents may be developed as novel therapeutics.

## Background

The Acanthaceae family is used in many South and East Asia countries as the ethno pharmacological medicines. Some researchers have indicated that Acanthaceae possess antifungal, cytotoxic, anti-inflammatory, anti-pyretic, hepatoprotective, immunomodulatory, anti-platelet aggregation and anti-viral potential [1]. This family has about 35 genera and 304 species in China. *Justicia* is the largest genus. *J. procumbens* is a commonly used traditional herbal medicine embodied in Chinese Pharmacopoeia 1977 version. The entire plant has long been used to treat laryngeal inflammation, pain, and cancer in China [2]. There are abundant resources in south China.

**Fig. 1:**
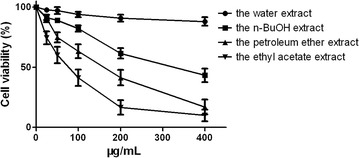
The inhibition effect of four extracts on A549 cells. Each value represents the mean ± SD of five separate experiments

In the preliminary study, the EtOH extract of *J. procumbens* was suspended in water and partitioned with petroleum ether, ethyl acetate, and *n*-BuOH. The results of the MTT assay showed that the ethyl acetate extract had a stronger cytotoxic activity against the human lung epithelial cell A549 than the other extract (Fig. 1). Therefore, it was important to identify the chemical constituents in the ethyl acetate extract.

In the past years, diverse compounds have been isolated from *J. procumbens*, mainly arylnaphthalide and diarylbutane lignans, and their glycosides [3, 4]. However, these individual chemical studies were characterized by long span of time, accidentally discover, and subsection. HPLC–MS combining the selected chromatographic column with quantitative analysis could provide the whole landscape of characteristic chromatogram from plants [5, 6]. By matching with this characteristic chromatogram, a complete and systematic phytochemistry study could be carried out without missing any of potential active compounds.

In order to reveal effective substances in the ethyl acetate extract of *J. procumbens*, a HPLC–ESI–QTOF–MS analysis method has been developed. To improve liquid chromatographic resolution, an ether-linked phenyl column was used. The structure of the unidentified isomeride and novel compound were characterized by NMR. Finally, 23 lignans were identified. Compound 12 is novel. Compounds 1, 2, and 13 were detected for the first time. The simultaneous analysis of the lignans present in *J. procumbens* using HPLC–DAD–ESI–MS has not been reported.

## Experimental methods

### Materials and chemicals

The plant materials of *J. procumbens* were collected from Wuchang district, Hubei province of China in 2014. They were authenticated by Prof. Keli Chen, Hubei University of Chinese Medicine. All the voucher specimens (JC-2014-ZYYDX) were deposited in the pharmaceutical college.

The human lung epithelial cell A549 were obtained from China Center for Type Culture Collection (Wuhan, China). Roswell Park Memorial Institute (RPMI) 1640 medium, fetal bovine serum (FBS), and penicillin–streptomycin were purchased from Gibco Corporation (New York, USA). MTT (thiazolyl blue tetrazolium bromide) and dimethyl sulfoxide (DMSO) were purchased from Sigma-Aldrich Corporation (St. Louis, MO, USA).

HPLC–MS grade acetonitrile was purchased from Fisher Scientific UK (Loughborough, UK). All other analytical grade reagents were purchased from Shanghai Chemical Reagent Corporation of China Medicine Group (Shanghai, China). The water for HPLC analysis was prepared using a Milli-Q SP Regent Water system (Millipore, USA).

### Extraction and isolation

The entire plants were dried at room temperature for 1 week and then ground to fine powder using a mechanical grinder. The powdered sample (30 kg) was immersed in 75% EtOH (240 L). After the evaporation of EtOH under reduced pressure at 50 °C, the residues (8.8 L) were successively partitioned using petroleum ether (590 g), ethyl acetate (240 g), and *n*-BuOH (360 g). The ethyl acetate extract (200 g) was chromatographed on silica gel using a mixture of CHCl_3_–MeOH (50:1 to 1:1) and on Sephadex LH-20 using a mixture of CHCl_3_–MeOH and MeOH. Eight compounds, 5-methoxy-4,4′-di-*O*-methylsecolariciresinol-9′-monoacetate (16 mg), justicidinoside B (117 mg), justicidinoside C (182 mg), procumbenoside B (105 mg), procumbenoside H (79 mg), justicidin B (636 mg), chinensinaphthol methyl ether (217 mg), and neojusticin B (93 mg) were obtained. The structures of these compounds were elucidated from their MS and NMR spectral data.

### Assays for cytotoxic activity

#### Cell culture

The human lung epithelial cells A549 grown adhesively in RPMI 1640 medium supplemented with 10% fetal bovine serum (FBS), 100 U/ml penicillin and 100 U/ml streptomycin. Cells were cultured at 37 °C in 5% CO_2_ humidified atmosphere. The cell passage was carried out every 2–3 days.

#### MTT assay

Cells were seeded in 96-well plated at a density of 1 × 10^4^ cells/ml in a volume of 100 μl/well. After cells adhesion was observed, the spent medium was removed and replaced with 100 μl of fresh medium doped with different concentrations of the four extracts (25, 50, 100, 200, and 400 μg/ml) for 48 h in quintuplicate. Subsequently, 20 μl of 5 mg/ml MTT solution were added followed by incubation for an additional 4 h. Then, the supernatant was discarded and 100 μl of DMSO was added each well. The absorbance was measured at 570 nm with plate reader use (Biotek, Cytation 3). The results were expressed as percentage viability.

#### Standard and sample preparation

The standard stock solutions of justicidinoside B (0.42 mg/ml), justicidinoside C (0.59 mg/ml), procumbenoside H (0.37 mg/ml), justicidin B (0.92 mg/ml), chinensinaphthol methyl ether (0.34 mg/ml), and neojusticin B (0.44 mg/ml) were prepared in methanol and stored at 4 °C. Appropriate concentrations were diluted for preparing calibration curves and the mixed standard solution. The solutions were filtered through a 0.45 μm membrane prior to injection.

The ethyl acetate extract powder (40 mg) was accurately weighed and placed in a 250 ml capped conical flask. Then, 100 ml methanol was added, and the mixture was extracted using an ultrasonic bath (50 Hz) for 30 min. The extract was filtered through a 0.45 μm membrane filter. Finally, 10 μl of the sample was injected into an HPLC instrument for analysis.

#### HPLC–DAD–ESI–QTOF–MS conditions

The HPLC analysis was performed using an Agilent 1260 Infinity system (Agilent, America). Chromatographic separations of the analytes were carried out using a Synergi Polar-RP 80 A column (4.6 mm × 250 mm, 4 μm particle size) obtained from Phenomenex at 30 °C. The mobile phase consisted of water (solvent A) and acetonitrile (solvent B); the gradient program was as follows: 0 min 15% B, 130 min 35% B, and 175 min 45% B. The flow rate was 1.0 ml/min, and the injection volume was 10 μl. The on-line UV spectra were recorded in the range 190–400 nm.

The QTOF–MS spectra were acquired using a micrOTOF-Q mass spectrometer equipped with an ESI source (Bruker Daltonics, Bremen, Germany). The optimized MS operating conditions were as follows: capillary voltage 4500 V, nebulizer gas pressure 0.8 bar, drying gas flow rate 8 l/min, dry gas heater temperature 200 °C in the positive ion mode (ESI^+^). The mass scan range was set at *m*/*z* 50–1600.

#### NMR conditions

The ^1^H-NMR spectra of justicidinoside B, justicidinoside C, procumbenoside B, procumbenoside H, justicidin B, chinensinaphthol methyl ether, and neojusticin B were recorded using Bruker Avance III 600 MHz instrument. These arylnaphthalenes were dissolved in CD_3_OD.

The ^1^H-NMR, ^13^C-NMR, ^1^H-^1^H COSY, HSQC, and HMBC spectra of 5-methoxy-4,4′-di-*O*-methylsecolariciresinol-9′-monoacetate were recorded using Bruker Avance III 800 MHz instrument. This dibenzylbutane was dissolved in CDCl_3_.

## Results and discussion

### Evaluation of cytotoxic effect

A549 cells were cultured in a medium containing different concentrations of the four extracts for 48 h. The cell viabilities were determined by MTT assay as shown in Fig. 1. We found that cell growth was inhibited in the following order: the ethyl acetate extract > the petroleum ether extract > the *n*-BuOH extract > the water extract. The ethyl acetate extract had a stronger cytotoxic activity than the other extracts and dose–effect relationship was observed. The IC_50_ of this extract was 66.93 μg/ml.

### Screening high performance liquid chromatography

A good chromatographic separation of the constituents in the ethyl acetate extract of *J. procumbens* was achieved using a reverse-phase column and a gradient elution with a mixture of water and acetonitrile. The hydrophilic and hydrophobic lignans were determined simultaneously using an ether-linked phenyl column (Synergi Polar-RP 80 A). These compounds showed a low resolution on a C18 column. The ratio of acetonitrile in the mobile phase was increased to 100% after the gradient program, none peak was observed. This result indicated that all of compounds in the ethyl acetate extract have been detected in 175 min. The ionization mode was very influential on the number of detected chemical substances in the ethyl acetate extract of *J. procumbens*. The positive ionization mode was the most favorable to identify chemical substances as it clearly provided a higher sensitivity.

**Fig. 2:**
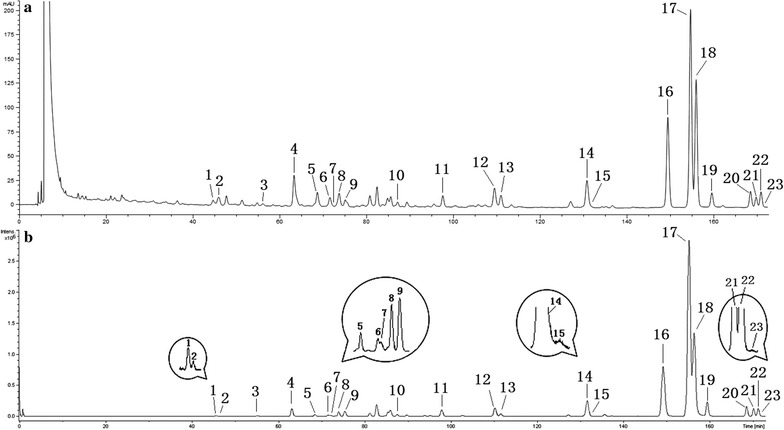
HPLC–DAD–ESI–QTOF/MS chromatogram of the ethyl acetate extract of *J. procumbens*: **a** UV chromatogram obtained at 205 nm, **b** TIC chromatogram detected in the positive ion mode

**Fig. 3:**
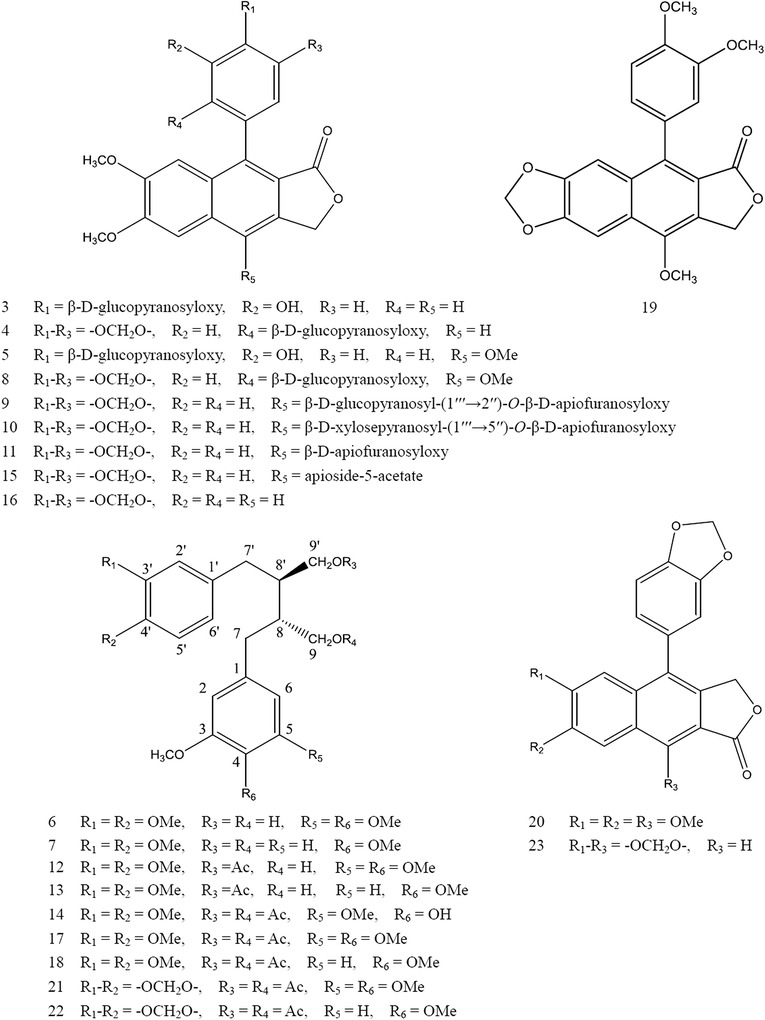
Structures of compounds 3–23

More than 23 peaks were detected in the MS total ion current (TIC) chromatogram in the positive ion mode (Fig. 2). The exact molecular weight and MS fragmentation data of the 23 compounds are summarized in Table 1. Their structures were deduced by carefully studying the HRMS, and NMR spectral data and by comparing with the library of compounds obtained from the plant of *Justicia* genus. The 23 compounds included 11 dibenzylbutanes and 12 arylnaphthalenes (Fig. 3).

**Table 1:** Characterization of 23 compounds in the ethyl acetate extract of *J. Procumbens* by HPLC–DAD–ESI–MS

	RT (min)	λ_max_ (nm)	Formula	Parent ion	Exact molecular weight	Fragmentation	Identification
1	45.5	–	C_29_H_42_O_12_	[M+Na]^+^	605.2578	421.2211, 403.2114, 385.2000, 181.0855, 151.0759	Glycoside of compound 6
2	45.8	–	C_28_H_40_O_11_	[M+Na]^+^	575.2471	391.2113, 355.1881, 165.0916, 151.0760	Glycoside of compound 7
3	57.9	262	C_26_H_26_O_11_	[M+Na]^+^	537.1351	353.1012, 335.0906, 307.0968	Procumbenoside L
4	63.1	257	C_27_H_26_O_12_	[M+Na]^+^	565.1319	381.0953, 337.1061 323.0912, 307.0951	Justicidinoside C
5	67.8	265	C_27_H_28_O_12_	[M+Na]^+^	567.1475	383.1113, 369.1001, 365.0987, 339.1217, 337.1064	Procumbenoside K
6	71.5	–	C_23_H_32_O_7_	[M+Na]^+^	443.2026	403.2015, 385.2002, 247.0938, 217. 0828, 181.0851, 151.0760	5-methoxy-4,4′-di-*O*-methylsecolariciresinol
7	73.3	–	C_22_H_30_O_6_	[M+Na]^+^	413.1920	373.1999, 355.1888, 217.0831, 165.0911, 151.0754	Secoisolariciresinol dimethyl ether
8	74.0	263	C_28_H_28_O_13_	[M+Na]^+^	595.1420	411.1063, 367.1164, 337.1056	Justicidinoside B
9	75.4	264	C_32_H_34_O_16_	[M+H]^+^	675.1892	513.1393, 381.0951	Procumbenoside B
10	87.5	265	C_31_H_32_O_15_	[M+H]^+^	645.1806	513.1393, 381.0952	Procumbenoside H
11	97.8	263	C_26_H_24_O_11_	[M+H]^+^	513.1395	381.0951	Tuberculatin
12	110.1	279	C_25_H_34_O_8_	[M+Na]^+^	485.2140	403.2097, 385.1991, 181.0853, 151.0757	5-methoxy-4,4′-di-*O*-methylsecolariciresinol-9′-monoacetate
13	111.5	282	C_24_H_32_O_7_	[M+Na]^+^	455.2024	395.1824, 373.1992, 355.1904, 165.0904, 151.0758	Secoisolariciresinol dimethyl ether monoacetate
14	131.4	264	C_26_H_34_O_9_	[M+Na]^+^	513.2113	371.1845, 339.1584, 233.0795, 217.0843, 177.0910, 167.0702, 151.0758	Justin C
15	131.5	–	C_28_H_26_O_12_	[M+H]^+^	555.1495	513.1407, 381.0947	Diphyllin apioside-5-acetate
16	149.0	258	C_21_H_16_O_6_	[M+Na]^+^	387.0824	335.0899, 321.0749	Justicidin B
17	155.0	201, 228, 278	C_27_H_36_O_9_	[M+Na]^+^	527.2250	505.2422, 445.2235, 403.2104, 385.1997, 247.0944, 217.0824, 195.1007, 181.0853, 177.0912, 151.0752	5-methoxy-4,4′-di-*O*-methylsecolariciresinol diacetate
18	156.2	201, 230, 280	C_26_H_34_O_8_	[M+Na]^+^	497.2147	355.1899, 325.1806, 313.1792, 269.1535, 217.0839, 195.1005, 165.0902, 151.0753	Secoisolariciresinol dimethyl ether diacetate
19	159.2	262	C_22_H_18_O_7_	[M+H]^+^	395.1108	365.1015, 351.0859, 319.0972	Chinensinaphthol methyl ether
20	168.3	262	C_22_H_18_O_7_	[M+H]^+^	395.1110	365.1007	Neojusticin B
21	169.9	282	C_26_H_32_O_9_	[M+Na]^+^	511.1916	387.1791, 369.1693, 247.0931, 201.0521, 195.1009, 181.0851, 151.0759	(−)-dihydroclusin diacetate
22	171.0	284	C_25_H_30_O_8_	[M+Na]^+^	481.1809	339.1580, 201.0527, 177.0904, 165.0910, 151.0753, 135.0449	2,3-demethoxysecisolintetralin acetate
23	171.7	–	C_20_H_12_O_6_	[M+H]^+^	349.0648	305.0795	Justicidin E

The compound 5-methoxy-4,4′-di-*O*-methylsecolariciresinol-9′-monoacetate (**12**) was identified for the first time. This is the first report that two glycosides (**1** and **2**) of secoisolariciresinol dimethyl ether and 5-methoxy-4,4′-di-*O*-methylsecolariciresinol, one monoacetate (**13**) of secoisolariciresinol dimethyl ether were detected. Further structural studies of the three compounds are underway.

### Identification of dibenzylbutanes

Dibenzylbutane lignans are molecules with two benzene rings in their structure that can be divided into two subgroups. The first fragmentation stage is the cleavage of the glycosidic or acetic bound to yield the *m*/*z* of the dibenzylbutane lignan and the neutral mass loss of sugar or acetoxy molecules. The second characteristic fragmentation stage is the bond cleavage between C8 and C8′. The fragmentations of this stage are helpful to identify the specific dibenzylbutane lignans directly.

**Fig. 4:**
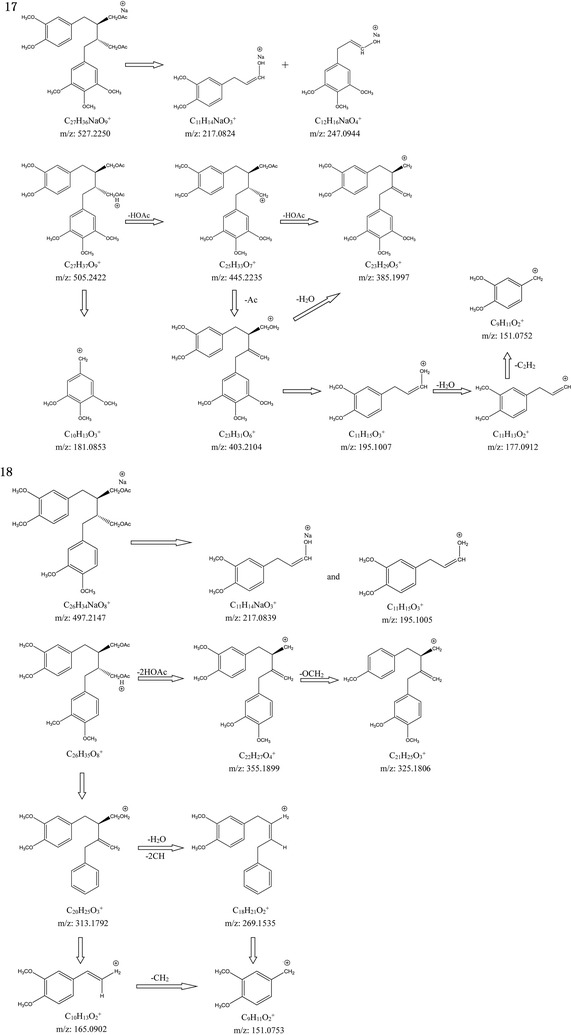
Proposed fragmentation pattern of compounds 17 and 18

Compounds 18, 13, 7, and 2 showed similar characteristic features in the mass spectra. All of them showed fragment ions nearby *m/z* 355.1899, 165.0902, and 151.0753 (Table 1). Compound 18 showed a [M+Na]^+^ ion at *m/z* 497.2147, and its chemical formula is C_26_H_34_O_8_. The MS spectrum showed two main fragments at *m/z* 355.1899 (loss of diacetate) and *m/z* 151.0753 (C_9_H_11_O_2_^+^). The characteristic fragment ion at *m/z* 217.0839 was attributed to the bond cleavage between C8 and C8′. The fragment pathways are shown in Fig. 4. This compound was identified as secoisolariciresinol dimethyl ether diacetate by matching with the library [3].

Compound 13 showed a [M+Na]^+^ ion at *m/z* 455.2024, and its chemical formula is C_24_H_32_O_7_. Two ions at *m/z* 395.1824 [M+Na-HOAc]^+^, and 373.1992 [M+H-HOAc]^+^ were observed. This compound was tentatively identified as secoisolariciresinol dimethyl ether monoacetate.

Compound 7 showed a [M+Na]^+^ ion at *m/z* 413.1920, and its chemical formula is C_22_H_30_O_6_. Two peaks were observed at *m/z* 373.1999 [M+H-H_2_O]^+^ and 355.1888 [M+H-2H_2_O]^+^. The structure was deduced as secoisolariciresinol dimethyl ether [7].

Compound 2 showed a [M+Na]^+^ ion at *m/z* 575.2471 (C_28_H_40_NaO_11_^+^) and the aglycone fragment ion at *m/z* 391.2113, [M+H-162]^+^, indicating a loss of hexose from the parent ion. The characteristic ions shown in Table 1 confirm that the aglycone was compound 7. However, the glycosilation position and the structure of hexose could not be obtained by MS.

Compounds 17, 12, 6, and 1 showed similar characteristic features in the mass spectra. The mass spectrum of compound 17 showed a molecular ion peak at *m/z* 527.2250 [M+Na]^+^, and its chemical formula is C_27_H_36_O_9_. The most abundant fragment peak at *m/z* 385.1997 was produced by the loss of diacetate from [M+H]^+^ ion. The formation of a characteristic fragment ion at *m/z* 247.0944 with chemical formula C_12_H_16_NaO_4_^+^ and another ion at *m/z* 217.0824 with chemical formula C_11_H_14_NaO_3_^+^ can be attributed to the bond cleavage between C8 and C8′. Other characteristic fragment ions were observed at *m/z* 445.2235, 403.2104, 195.1007, 181.0853, 177.0912, and 151.0752 (Fig. 4 and Table 1). Some similar patterns of mass spectra were observed for compounds 1, 6, and 12. They showed similar fragment pathways. The structure of this compound was confirmed as 5-methoxy-4,4′-di-*O*-methylsecolariciresinol diacetate by comparing with the Ref. [3].

Compound 12 produced a [M+Na]^+^ ion at *m/z* 485.2140, and its chemical formula is C_25_H_34_O_8_. The main peak was observed at *m*/*z* 403.2097 [M+H-HOAc]^+^. It is the same as 5-methoxy-4,4′-di-*O*-methylsecolariciresinol monoacetate. But the position of acetoxy wasn’t confirmed by MS.

Compound 6 showed a [M+Na]^+^ ion at *m/z* 443.2026, and its chemical formula is C_23_H_32_O_7_. The characteristic fragment ions were observed at *m/z* 403.2105 [M+H-H_2_O]^+^ and 385.2002 [M+H-2H_2_O]^+^. The structure was deduced as 5-methoxy-4,4′-di-*O*-methylsecolariciresinol [8].

Compound 1 showed a [M+Na]^+^ ion at *m/z* 605.2578 (C_29_H_42_NaO_12_^+^) and the aglycone fragment ion at *m/z* 421.2211, [M+H-162]^+^, indicating a loss of hexose from the mother ion. The characteristic fragments of aglycone were in accordance with compound 6 (Table 1). However, it was not possible to establish the exact glycosilation position (at C9 or C9′) and the structure of hexose.

**Fig. 5:**
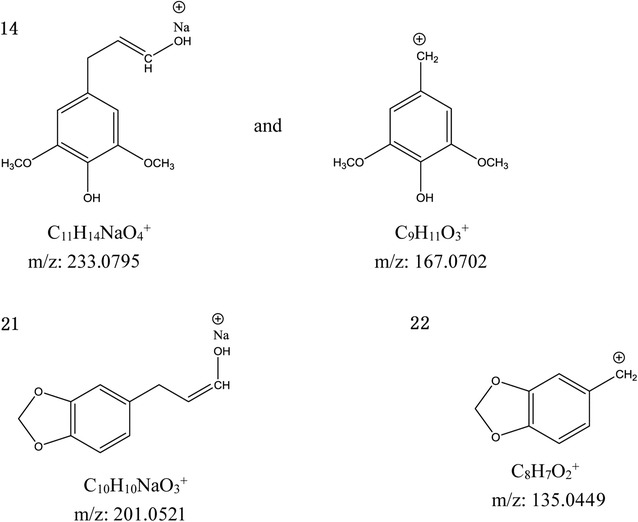
Pivotal fragment ions of compounds 14, 21, and 22

Compound 14 showed a [M+Na]^+^ ion at *m/z* 513.2113, and its chemical formula is C_26_H_34_O_9_. The main peaks were observed at 233.0795, and 167.0702 (Fig. 5). The fragmentations showed peaks at *m/z* 371.1845 [M+H-2HOAc], 339.1584 [M+H-2HOAc-CH_2_-H_2_O], 217.0843, 177.0910, and 151.0758. This compound was confirmed as Justin C [3].

Compound 21 showed a [M+Na]^+^ ion at *m/z* 511.1916, and its chemical formula is C_26_H_32_O_9_. The characteristic fragment ions at *m/z* 387.1791 [M+H-HOAc-Ac]^+^ and 369.1693 [M+H-2HOAc]^+^ indicated the loss of diacetate from the parent ion. The main ion was observed at *m/z* 201.0521 (C_10_H_10_NaO_3_^+^) (Fig. 5). The other characteristic fragment ions at *m/z* 247.0931, 195.1009, 181.0851, and 151.0759 were the same as those in compound 17 (Fig. 4). The structure was deduced as (−)-dihydroclusin diacetate [3].

Compound 22 showed a [M+Na]^+^ ion at *m/z* 481.1809, and its chemical formula is C_25_H_30_O_8_. The ion at *m/z* 339.1580 was attributed to the loss of diacetate from the parent ion. The characteristic fragment ions at *m/z* 201.0527, 177.0904, 165.0910, 151.0753, and 135.0449 were also observed in compounds 17 and 19. The structure was identified as 2,3-demethoxysecisolintetralin acetate [9].

### Identification of arylnaphthalenes

Arylnaphthalene lignans have the phenyl-naphthyl skeleton. The following steps are mandatory to obtain characteristic fragment: First, the cleavage of the glycosidic bonds to the aglycone take place to yield the *m*/*z* of the arylnaphthalene lignan without the neutral mass of the released sugars; second, when all glycosidic bonds are broken, the fragmented with the aglycone *m*/*z* is obtained; finally, characteristic fragmentations are showed the loss of CO_2_, CH_2_, and H_2_O groups from aglycone ion.

**Fig. 6:**
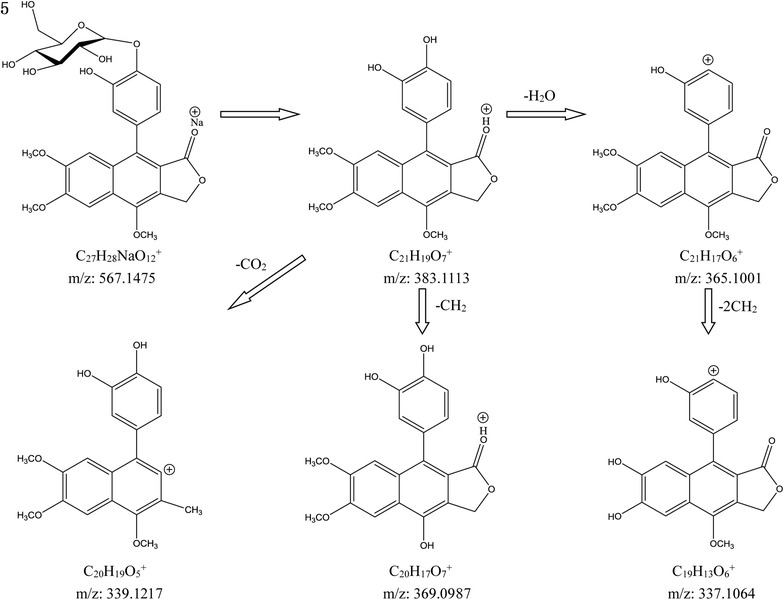
Characteristic fragment ions of compound 5

Compound 3 with the [M+Na]^+^ ion at *m/z* 537.1351 (C_26_H_26_NaO_11_^+^) showed the neutral loss of 162 Da (glucosyl residue), producing a fragment ion at *m/z* 353.1012 (C_20_H_17_O_6_^+^). Compound 5 showed a [M+Na]^+^ ion at *m/z* 567.1475 (C_27_H_28_NaO_12_^+^) that eliminated a glucosyl residue of 162 Da to produce an ion at *m/z* 383.1113 (C_21_H_19_O_7_^+^). Compound 11 showed a [M+H]^+^ ion at *m/z* 513.1395 (C_26_H_25_O_11_^+^) and the aglycone fragment ion at *m/z* 381.0951 (− 132 Da, an apioside residue). Compound 15 showed a [M+H]^+^ ion at *m/z* 555.1495 (C_28_H_27_O_12_^+^) and produced an important fragment ion at *m/z* 513.1407 (the loss of acetate moiety). The other characteristic fragments were the same as those in compound 11. Compound 23 produced a [M+H]^+^ ion at *m/z* 349.0648 (C_20_H_13_O_6_^+^). The fragmentations of compounds 3, 5, 11, 15, and 23 showed the loss of CO_2_, CH_2_, and H_2_O groups from parent ion or aglycone ion (Compound 5 as an example is shown in Fig. 6). None of the isomers of the five compounds were observed in the high-resolution mass spectrum and bibliography, unique arylnaphthalenes in *J. procumbens.* Compounds 3, 5, 11, 15, and 23 were identified as procumbenoside L, procumbenoside K, tuberculatin, diphyllin apioside-5-acetate, and Justicidin E by matching with the literatures [10–15].

Some other arylnaphthalenes in *J. procumbens* have the same molecular weight and the same fragment ion patterns, such as justicidinoside C and cleistanthin B [14, 15]. Further, the stereochemistry of compounds 4, 8, 9,10, 16, 19, and 20 were determined by ^1^H-NMR and compared to the literature. Compound 4 showed a molecular ion [M+Na]^+^ peak at *m/z* 565.1319 (C_27_H_26_NaO_12_^+^) and produced a product ion at *m/z* 381.0953 (the loss of glucosyl residue). Compound 8 showed a [M+Na]^+^ peak at *m/z* 595.1420 (C_28_H_28_NaO_13_^+^); a fragmentation at *m/z* 411.1063 (C_22_H_19_O_8_^+^) was attributed to the elimination of a glucosyl residue. Compound 9 showed a [M+H]^+^ peak at *m/z* 675.1892 (C_32_H_35_O_16_^+^). The parent ion produced a peak at *m/z* 513.1393 (C_26_H_25_O_11_^+^) via the loss of a glucosyl residue (162 Da); further fragmentation of this ion showed a peak at *m/z* 381.0951 (C_21_H_17_O_7_^+^) due to the loss of an apiosyl residue (132 Da). Compound 10 produced a [M + H]^+^ peak at *m/z* 645.1806 (C_31_H_33_O_15_^+^); the fragment at *m/z* 513.1393 (C_26_H_25_O_11_^+^) can be attributed to the cleavage of a xylosyl residue (132 Da) and 381.0951 (C_21_H_17_O_7_^+^) due to the removal of an apiosyl residue from C_26_H_25_O_11_^+^. Compound 16 showed a [M+Na]^+^ ion at *m/z* 387.0824 (C_21_H_16_NaO_6_^+^). Compound 19 showed a [M+H]^+^ ion at *m*/*z* 395.1108 (C_22_H_19_O_7_^+^). Compound 20 showed a [M+H]^+^ ion at *m*/*z* 395.1110 (C_22_H_19_O_7_^+^), too. In the ^1^H-NMR spectra of 4, 8, 9, 10, 16, 19, and 20 typical signals due to the arylnaphthalene lignan, were observed along with the signals owing to the sugar portion (Tables 2 and 3). Compounds 4, 8, 9, 10, 16, 19 and 20 were tentatively identified as justicidinoside C, justicidinoside B, procumbenoside B, procumbenoside H, justicidin B, chinensinaphthol methyl ether, and neojusticin B, respectively, by comparing with the literatures [3, 16–18].

**Table 2:** ^1^H-NMR data for compounds 4, 8, 9, and 10 (CD_3_OD)

Position	4	8	9	10
	δ_H_ (*J* in Hz)	δ_H_ (*J* in Hz)	δ_H_ (*J* in Hz)	δ_H_ (*J* in Hz)
4	7.73 (s)			
5	7.26 (s)	7.48 (s)	7.60 (s)	7.66 (s)
8	6.95 (s)	6.91 (s)	6.96 (s)	7.05 (s)
12	5.30 (2H, s)	5.53 (2H, s)	5.53 (d, 14.7) 5.42 (d, 14.7)	5.56 (d, 14.8) 5.48 (d, 14.1)
2′	6.53 (s)	6.49 (s)	6.71 (d, 1.2)	6.78 (d, 2.2)
5′	7.02 (s)	7.01 (s)	6.86 (d, 7.8)	6.95 (d, 7.7)
6′			6.68 (dd, 1.2, 7.9)	6.78 (dd, 1.7, 7.0)
3′-OCH_2_O-4′	5.94 (s) 5.91 (s)	5.93 (s) 5.90 (s)	5.96 (s) 5.94 (s)	6.05 (s) 6.04 (s)
4-OCH_3_		4.07 (3H, s)		
6-OCH_3_	3.89 (3H, s)	3.89 (3H, s)	3.94 (3H, s)	4.02 (s)
7-OCH_3_	3.66 (3H, s)	3.66 (3H, s)	3.63 (3H, s)	3.73 (s)
1′′	4.62 (d, 7.8)	4.59 (d, 8.0)	5.74 (d, 1.9)	5.49 (d, 3.6)
2′′	2.78 (t, 7.9)	2.80 (t, 8.6)	4.68 (d, 2.3)	4.58 (d, 3.8)
3′′	3.21 (m)	3.21 (m)		
4′′	3.07 (t, 9.4)	3.08 (t, 9.4)	4.19 (d, 9.7) 3.81 (d, 9.7)	4.35 (d, 9.6) 3.99 (d, 10.7)
5′′	3.21 (m)	3.21 (m)	4.50 (dd, 11.6) 3.64 (dd, 11.6)	4.01 (d, 10.8) 3.79 (d, 9.6)
6′′	3.49 (2H, dd, 5.8, 11.9)	3.50 (2H, dd, 5.8, 11.9)		
1′′′			4.52 (d, 7.8)	4.34 (d, 7.2)
2′′′			3.14 (m)	3.29 (t, 11.0)
3′′′			3.25 (m)	3.39 (t, 8.4)
4′′′			3.31 (t, 9.0)	3.54 (m)
5′′′			3.41 (m)	3.92 (dd, 5.2, 10.9) 3.24 (dd, 9.4, 10.7)
6′′′			3.69 (dd, 2.1, 11.6) 3.48 (dd, 4.8, 11.8)

**Table 3:** ^1^H-NMR data for compounds 16, 19, and 20 (CD_3_OD)

Position	16	20	Position	19
	δ_H_ (*J* in Hz)	δ_H_ (*J* in Hz)		δ_H_ (*J* in Hz)
4	7.72 (s)		4	
5	7.23 (s)	7.41 (s)	5	7.35 (s)
8	7.01 (s)	7.00 (s)	8	6.99 (s)
11		5.17 (2H, s)	11	
12	5.39 (2H, s)		12	5.41 (2H, s)
2′	6.86 (d, 1.5)	6.85 (d, 1.6)	2′	6.91 (s)
5′	6.94 (d, 7.9)	6.98 (d, 8.0)	5′	7.03 (s)
6′	6.80 (dd, 1.5, 7.8)	6.82 (dd, 1.6, 8.1)	6′	6.76 (dd, 1.6, 8.0)
3′-OCH_2_O-4′	5.98 (s)	6.06 (s)	3′-OCH_3_	3.86 (3H, s)
	5.94 (s)	6.00 (s)	4′-OCH_3_	3.70 (3H, s)
4-OCH_3_		4.31 (3H, s)	4-OCH_3_	4.03 (3H, s)
6-OCH_3_	3.85 (3H, s)	3.95 (3H, s)	6-OCH_2_O-7	6.17 (2H, d)
7-OCH_3_	3.64 (3H, s)	3.72 (3H, s)		

### Structural analysis of novel compound 12

**Fig. 7:**
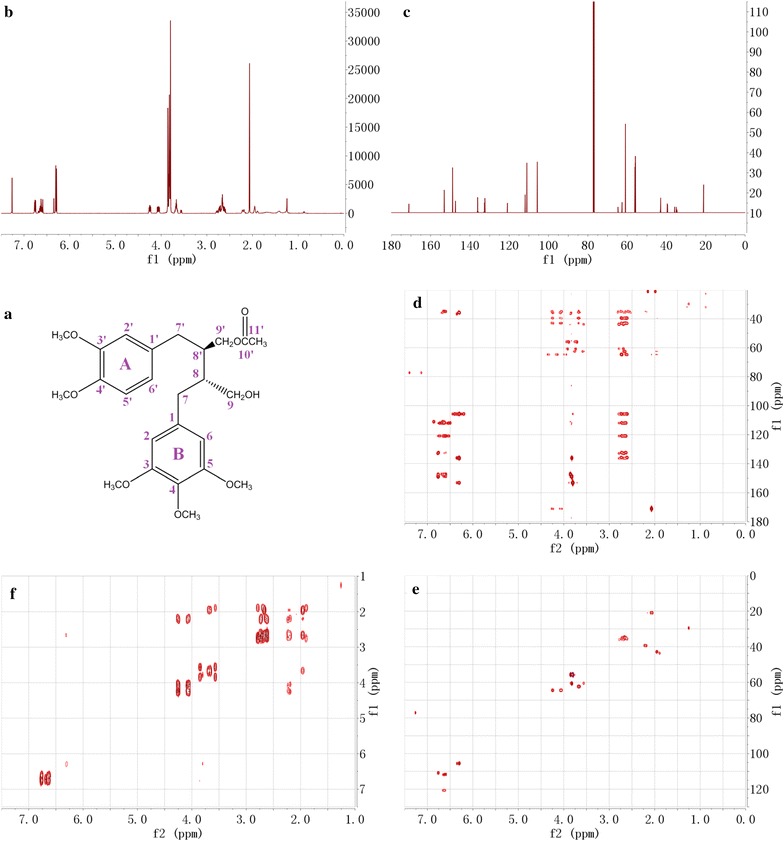
NMR spectra of compound 12: **a** chemical structure, **b** ^1^H-NMR spectrum, **c** ^13^C-NMR spectrum, **d** HMBC spectrum, **e** HSQC spectrum, **f** ^1^H-^1^H COSY spectrum

Compound 12 chemical formula is C_25_H_34_O_8_, which was deduced from the positive-ion HR-ESI–MS (*m*/*z* 485.2140 [M+Na]^+^). The ^1^H-NMR and ^13^C-NMR spectra (Table 4 and Fig. 7) showed signals of acetate hydrogens at δ 2.07 (3H, s, H-11′) and carbons at δ 21.11 (1C, C-11′), 171.05 (1C, C-10′). This acetate was located at the position C-9′ based on the HMBC spectrum correlation between H-9′ and C-10′. The ^1^H-NMR spectrum also showed signal of five aromatic hydrogens at δ 6.29 (1H, d, H-2 or 6), 6.30 (1H, d, H-2 or 6) of ring B and an ABX system of ring A at δ 6.63 (1H, m, H-2′), 6.76 (1H, m, H-5′), 6.66 (1H, m, H-6′). The corresponding carbons and methoxyls signals of benzene rings could be confirmed by the HSQC and HMBC spectra. According to the ^1^H-^1^H COSY spectrum of butyl portion, δ 4.06 (1H, ddd, H-9′a) and δ 4.24 (1H, ddd, H-9′b) showed correlation with the hydrogen signal at δ 2.21 (1H, m, H-8′), δ 2.21 (1H, m, H-8′) showed correlation with the hydrogen signal at δ 2.65 (1H, m, H-7′a) and δ 2.70 (1H, m, H-7′b), δ 3.67 (2H, dtt, H-9a, 9b) showed correlation with the hydrogen signal at δ 1.95 (1H, m, H-8), δ 1.95 (1H, m, H-8) showed correlation with the hydrogen signal at δ 2.61 (1H, m, H-7a) and δ 2.75 (1H, m, H-7b). The corresponding carbons signals of butyl could be confirmed by HSQC spectrum. The above mentioned spectroscopic data suggested that novel compound 12 is 2-(3,4-dimethoxybenzyl)-4-hydroxy-3-(3,4,5-trimethoxybenzyl)butyl acetate, which we named 5-methoxy-4,4′-di-*O*-methylsecolariciresinol-9′-monoacetate [3].

**Table 4:** ^13^C (200 MHz) and ^1^H-NMR (800 MHz) data for compound 12 (CDCl_3_)

Position	δ_C_	δ_H_ (*J* in Hz)	Position	δ_C_	δ_H_ (*J* in Hz)
1	136.11		1′	132.40	
2	105.70	6.29 or 6.30 (d, 1.7)	2′	111.90	6.63 (m)
3	153.10		3′	147.33	
4	153.10		4′	148.84	
5	153.10		5′	111.04	6.76 (m)
6	105.70	6.29 or 6.30 (d, 1.7)	6′	120.91	6.66 (m)
7a 7b	35.83	2.61 (m)	7′a	34.99	2.65 (m)
		2.75 (m)	7′b		2.70 (m)
8	42.97	1.95 (m)	8′	39.59	2.21 (m)
9	62.59	3.67 (2H, dtt)	9′a	64.69	4.06 (ddd)
			9′b		4.24 (ddd)
3-OCH_3_	60.89	3.80 (3H, s)	10′	171.05	
4-OCH_3_	60.89	3.80 (3H, s)	11′	21.11	2.07 (3H, s)
5-OCH_3_	60.89	3.80 (3H, s)	3′-OCH_3_	55.83	3.86 (3H, s)
			4′-OCH_3_	56.03	3.83 (3H, s)

Atom numbering as indicated in Fig. 7

All assignments are based on ^1^H-^1^H COSY, HSQC, and HMBC data

### Quantification of the six standard lignans

**Fig. 8:**
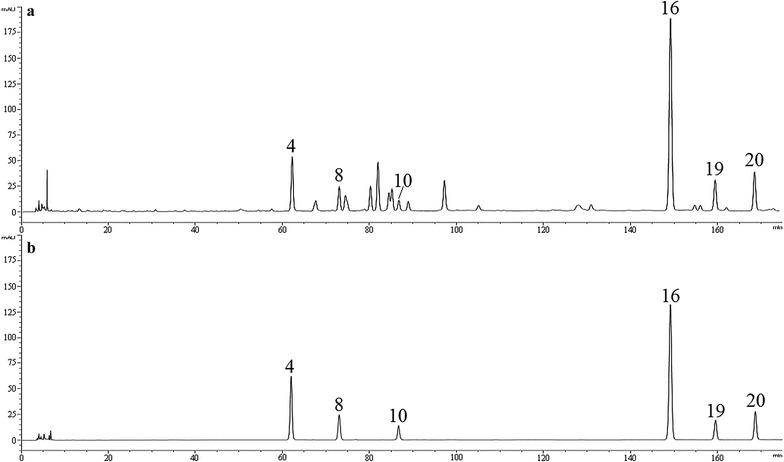
HPLC-UV chromatogram obtained at 260 nm: **a** sample, **b** six mixed standard

The maximum absorption peak of the arylnaphthalene lignans were around 260 nm. Therefore, the wavelength for the content determination of justicidinoside B, justicidinoside C, procumbenoside H, justicidin B, chinensinaphthol methyl ether, and neojusticin B were set at 260 nm. The content of procumbenoside B was not determined, because it was not separated with other compounds. The chromatograms of sample and mixed standard were shown in Fig. 8.

Standard curves were constructed by plotting the peak area against the corresponding concentration of the standard solutions. The limits of detection (LOD) and quantification (LOQ) were determined by diluting the working solutions when the signal-to-noise ratios (S/N) of about 3 and 10, respectively. The detailed descriptions of the curves were presented in Table 5.

**Table 5:** Linear regression data, LOD, and LOQ of six standard lignans

Standard	Regression equation	Linear range (μg/ml)	r^2^	LOD (μg/ml)	LOQ (μg/ml)
4	Y = 34.3060x − 2.2863	3.7–118	0.9999	0.021	0.071
8	y = 57.9005x − 3.3266	2.6–84	0.9999	0.013	0.044
10	y = 13.8900x − 5.3980	2.3–74	0.9998	0.043	0.142
16	y = 119.9074x − 40.5723	5.8–184	0.9999	0.005	0.017
19	y = 44.7432x − 18.4394	2.1–68	0.9998	0.017	0.057
20	y = 19.3973x − 23.6537	6.9–220	0.9998	0.034	0.113

In the regression equation Y = aX + b, X refers to the concentrations (μg/ml), Y is the peak area

The intra-day and inter-day assay precisions were respectively carried out on the mixture standard solution of six analytes six times a day and once a day for six sequential days, respectively. For the stability testing, the same sample was analyzed every 8 h within 48 h at the room temperature. The method repeatability was examined by the injection of six different sample, which were prepared with the same sample preparation procedure. The mean content of method repeatability were the content of six lignans in the ethyl acetate extract. Recovery was determined by adding an accurately known amount of the corresponding marker compounds to the known sample. The recovery rate and RSD for analytes at different concentrations were determined. All results were shown in Table 6.

**Table 6:** Precision, repeatability, stability, recovery, and content of six standard lignans

Standard	Precision (RSD %)		Repeatability (RSD %)	Stability (RSD %)	Recovery		Content^a^ (mg/g)
	Intra-day	Inter-day			Mean	RSD %	
4	0.86	1.63	1.78	1.39	101.49	1.66	34.70
8	0.99	1.37	1.93	0.88	101.20	1.97	8.93
10	1.10	1.85	1.96	1.16	98.63	2.00	10.10
16	0.64	0.99	1.31	0.73	100.51	1.53	28.36
19	0.92	1.72	1.81	1.57	99.37	1.95	12.22
20	0.51	0.94	1.35	0.94	99.04	1.83	29.05

^a^Content of six lignans in the ethyl acetate extract

The quality standard of *J. procumbens* has been included in the 1977 version of the Chinese Pharmacopoeia. However, this standard lacked the qualitative and quantitative analytical methods. This result will provide a scientific basis for the quality control of *J. procumbens*.

## Conclusions

Using a combination of liquid chromatography, high-resolution MS and NMR techniques, 23 compounds were identified, and four novel compounds (1, 2, 12, and 13) are reported for the first time. A HPLC–DAD–MS method was developed for the first time to analyze the chemical constituents of *J. procumbens* and detected the content of six lignans. The above results indicated that these compounds were the active chemical components responsible for the cytotoxic properties of *J. procumbens*.

Among 23 lignans, justicidin B, tuberculatin, and procumbenoside H have been proven potent cytotoxic activity against the Human LoVo and BGC-823 cell lines [18]. Chinensinaphthol methyl ether exhibited cytotoxic activity against the human leukemia K562 cell line [19]. Dihydroclusin diacetate had cytotoxic activity against the M12.C3.F3 and RAW264.7 murine cell lines [20]. Diphyllin apioside-5-acetate and neojusticin B showed cytotoxic against the cultured rabbit lung cell [14]. These reports also supported the cytotoxic activity of the ethyl acetate extract of *J. procumbens*.

This study identified the lignan constituents in the ethyl acetate extract of *J. procumbens*. The whole landscape of characteristic chromatogram data of lignans has been established. The complete and systematic phytochemistry studies are underway. After isolation of more pure constituents, the activities of individual compounds will be determined in the future, and the structure–activity relationship will be established.

This newly developed HPLC–DAD–ESI–MS method also provides a pathway to study the accumulation and distribution of secondary metabolites in *J. procumbens* and serves as a good strategy for the quality control of this plant.
